# A comparative analysis of the influence of contraceptive use and fertility desire on the duration of second birth interval in four sub-Saharan African countries

**DOI:** 10.1186/s12905-021-01486-z

**Published:** 2021-10-02

**Authors:** Rotimi Felix Afolabi, Adeniyi Francis Fagbamigbe, Martin Enock Palamuleni

**Affiliations:** 1 Department of Epidemiology and Medical Statistics, Faculty of Public Health, College of Medicine, University of Ibadan, Ibadan, Nigeria; 2 Population Studies and Demography Programme and Population and Health Research Entity, North-West University, Mmabatho, South Africa

**Keywords:** Fertility desire, Contraceptive use, Sub-Saharan Africa, Second birth interval, Survival analysis

## Abstract

**Background:**

Fertility decline characterised by inter-birth intervals remains rather slow or stall in many countries of sub-Saharan African (SSA). Non-adherence to optimal inter-birth intervals often occasioned by low prevalence of contraceptive use and high fertility desires often lead to poor maternal and child health outcomes. Additionally, information on the influence of contraception and fertility desire on interval between first and second births (SBI) is rarely available. This study therefore aimed to examine the influence of fertility desire and contraception on SBI among women in four SSA countries.

**Methods:**

We analysed cross-sectional data on women aged 15–49 years who participated in the recent Demographic and Health Surveys in DR Congo, Ethiopia, Nigeria and South Africa. Semi-parametric Cox proportional hazards regression was employed for the analysis at 5% significance level.

**Results:**

The median time to second birth was 34 months in DR Congo; 35 months, Nigeria; 42 months, Ethiopia; and 71 months, South Africa. About 70% of the women desired additional child(ren) and two-thirds have never used contraceptive in both Nigeria and DR Congo. The hazard of second birth was significantly lower among women who desired additional child(ren) compared to desired for no more child in DR Congo (aHR = 0.93; CI: 0.89–0.97), Ethiopia (aHR = 0.64; CI: 0.61–0.67) and South Africa (aHR = 0.51; CI: 0.47–0.55). Women who had never used contraceptive were 12%, 20% and 24% more likely to lengthen SBI than those who were current users in DR Congo, Nigeria and South Africa respectively. DR Congo and Nigerian women were about two times more likely to shorten SBI compared with their South African counterparts. Other significant determinants of SBI include ethnicity, rural residential, age and marital status at first birth, wealth and employment status.

**Conclusion:**

Findings showed differentials in the linkage between second birth interval and the desired fertility and contraception by country, demonstrating the importance of context. The contribution of these factors to second birth interval requires country context-specific attention if further decline in fertility and poor health outcomes associated with sub-optimal inter-birth interval is to be attained in SSA.

## Background

Even though fertility decline in most of the world’s high-fertility regions is remarkable, sub-Saharan African (SSA) remains the region with the highest total fertility rate (TFR). The SSA current TFR is estimated at 4.8 births per woman [1]. Besides, over the last two to three decades, fertility declines in SSA have been slow. Compared to 2000, though of varying magnitude region-wise, the current TFR among women declined from 6.1 to 4.4 in eastern, 6.2 to 5.2 in western, 6.5 to 5.6 in middle and 3.0 to 2.5 in the southern region of SSA [1–3]. Worrisome, four (Democratic Republic of Congo (DR Congo), Ethiopia, Nigeria, Tanzania) of the nine countries projected to contain over half of the increase in the world population by 2050 are in SSA [4]. The aforementioned should thus pose a challenge to policymakers in many countries of SSA to mitigate the impact of growing population, particularly of children and young adults.

Studies have linked the postponement of all higher-order births, including the second birth as a driver of SSA fertility decline [5, 6]. Usually, fertility transition commences at second birth and it is key to understanding subsequent childbearing experiences [7, 8]. Even with at least a fertility preference of two children, having second childbirth is a normative event for most SSA women. Therefore, the present study focused on the second birth interval (SBI), defined as the time elapsed between first and second childbirth.

The optimal length of SBI and other inter-birth intervals is usually three to five years, while at least 2 years is ideal before the next conception after birth [9–13]. Non-adherence to the ideal inter-birth intervals often lead to poor maternal and child health. Like any other birth interval, SBI could impact maternal and child health. Mahfouz and colleagues [13] submitted that inter-pregnancy interval less than 24 months is associated with a high risk of preterm birth and low birth weight; interval higher than 59 months, high likelihood of having stillbirth and pregnancy-induced hypertension. Similarly, other researchers [11, 14] have documented that a long birth interval (> 59 months) is attributed to poor maternal and child health outcomes. Unarguably, women experience sub-optimal birth interval in a region of high fertility such SSA.

Generally, the optimal inter-birth interval could be achieved through effective use of contraceptive. Many studies have shown that effective use of contraceptive impacted positively on inter-birth interval lengthening [15–19]. This invariably suggests that lengthened inter-birth intervals are a common feature of countries with higher contraceptive prevalence, and by extension lower TFR. Remarkably, women’s contraceptive behaviour has been linked to their desired fertility [20] which is positively associated with household size. Obviously, high desire for additional children may impede accelerated fertility decline [19]. But, low desired fertility may not alone necessarily resulted in a significant fertility decline [21]. Among others in the literature, socio-economic and demographic factors that could influence inter-birth interval include women age at first birth [16, 22], marital status [23, 24], education level [12, 19, 22, 25]; wealth status [26, 27]; residence [16, 28]; religion [29, 30], working status and parity [24, 31].

However, information on the effect of contraception and fertility desire on interval between first and second births is rarely documented. Yet there is evidence of inconsistency between desired fertility and contraceptive behaviour [20]. There is a need to explore the influence of contraceptive use and fertility desire on the duration of SBI. In light of this, the present study is aimed at investigating the impact of fertility desire and contraception on time to second childbirth among women of reproductive age in four sub-Saharan Africa countries, using the most recent Demographic and Health Survey (DHS) datasets. The outcome of this study may inform the promotion of family planning and subsequently address the challenge of unmet need for contraception in reducing maternal and child morbidity and mortality [15]. Additionally, it may inform context-specific strategies and policies aimed at containing the associated challenges of densely populated countries through advocacy for low fertility desire.

## Methods

### Study design and setting

Surveys (DHS) conducted in four SSA countries: DR Congo DHS (2013/14 DRCDHS), Ethiopia DHS (2016 EDHS), Nigeria DHS (2018 NDHS), South Africa DHS (2016 SADHS). The DHS is cross-sectional in design and provide population-based health indicators to assist policymakers and programme managers in designing and evaluating programs and strategies for improving the health of a country's population. The data, collected by trained field workers, contain self-reported information on the sexual and reproductive health history of the sampled women.

Currently, the four countries have diverse population sizes: DR Congo (90 million), Ethiopia (115 million), Nigeria (206 million) and South Africa (60 million); these constitute the largest population in middle, eastern, western, and southern regions of SSA [1], respectively. Of note, these countries are among the nine countries of the world projected to house more than 50% of the global population increase by 2050, except for South Africa [4]. The growth rates and TFR in DR Congo are respectively 3.5 and 6.2; Ethiopia, 2.7 and 4.3; Nigeria, 2.5 and 5.3; and South Africa, 1.1 and 2.3 [1]. Although South Africa fertility rate remains the lowest in sub-Saharan Africa, her observed fertility decline is still above the replacement level. Furthermore, these countries are classified as low-middle-income countries by the World Bank. Nigeria and South Africa are the two leading economic countries in the continent rated as middle-income; Ethiopia and DR Congo are rated as low-income countries.

In these countries, the DHS employs a stratified two-stage cluster sampling technique using the sampling frame containing the enumeration areas (EAs). In the first stage, clusters (otherwise known as EAs) are selected using a probability proportional-to-size approach per stratum. At the second stage, households are selected as the secondary sampling units using a systematic sampling per cluster. Women of childbearing age who reside in the respective countries were the study participants. A detailed description of the sampling design and strategies has been previously reported [32–35].

### Study population and variables

Of the 18,827, 15,683, 41,821, 8514 women aged 15–49 years who participated in the 2014 DRCDHS, 2016 EDHS, 2018 NDHS, 2016 SADHS respectively, 13,884, 10,114, 29,296, 6039 had had at least one birth and were included in the current study. These were the participants who had reported at least one singleton childbirth as of the survey date.

*Outcome variable* The dependent variable of interest was the time elapsed between first and second childbirth among women in the selected countries. Women who did not have second childbirth as at the time of the survey were right-censored and were coded 0; otherwise, 1 in the analysis.

*Independent variables* The key independent variables were contraceptive use and fertility desire. Use of contraceptive was derived from the questions asking women to indicate “whether they ever used anything or tried to delay or avoid getting pregnant” and their “current contraceptive use by method type”; this was categorised as “never-, former-, or current-use” of any means to delay/stop pregnancy/childbirth. Fertility desire, derived from the questions asking women to indicate whether they desired more children were re-categorised as “no-more, want, or undecided”. Based on existing empirical studies [16, 36], other covariates considered for the study were ethnicity, religion (Christianity, Islam, traditionalist/other), education (none, primary, secondary, tertiary), wealth index (low, middle, high), age (< 20, 20–24, ≥ 25 years) and marital status at-first-birth (never married, married before first-birth, married after first-birth), employment status (not working, working), first-birth sex (male, female) and first-birth survival (dead, alive). Of note, the wealth index variable, derived from the generated weighted factor score by principal component analysis as contained in the women recode file, was grouped into low, middle, and high wealth quintiles. It is a proxy measure of household socio-economic status due to the non-existence of information on household income. The term ethnicity indicated self-reported ethnic group in each of the country except South Africa, where information on skin colour was provided. Also, the information on religion was unavailable in the 2016 SADHS.

### Statistical data analysis

Survival analysis methods were used for the analysis. The “failure time” for women who have had second birth was the SBI. The “censored time” for the women without second birth yet was time since the first-birth and interview date. The Kaplan–Meier survival method was used to describe the women’s time to second birth while the log-rank test was employed to examine the association between SBI duration and the individual explanatory variables. Semi-parametric Cox proportional hazard (CPH) regression was thereafter used to evaluate the effect of contraceptive use and fertility desire on SBI amidst other variables controlled for, in each of the selected countries.

*Model expression* The CPH model can be written as:

1 $$h\left( {t_{i} } \right) = h_{0} \left( t \right)\ell^{{\sum\nolimits_{j = 1}^{p} {b_{j} x_{ji} } }} \,\mathop{\longrightarrow}\limits^{imlying}\,\ln \frac{{h\left( {t_{i} } \right)}}{{h_{0} \left( t \right)}} = \mathop \sum \limits_{j = 1}^{p} b_{j} x_{ji}$$

where ${b}_{j}$—jth coefficients of the explanatory variable *X*_*j*_, *p*—number of explanatory variables, ${h}_{0}\left(t\right)$—baseline hazard function such that ${\mathrm{h}\left(t\right)/h}_{0}\left(t\right)$—indicates the hazard ratio (HR), and the conditional probability of experiencing second childbirth within a short time interval (t, t + ∆t) having survived till time *t* is

2 $$h\left( t \right) = \mathop {{\text{lim}}}\limits_{\Delta t \to 0} \left\{ {\frac{{P\left( {t \le T \le t + \Delta t\left| T \right\rangle t} \right)}}{\Delta t}} \right\}$$

Usually, the relationship between hazard, H(t) and survival, S(t) functions is expressed as

3 $$S\left( t \right) = e^{ - H\left( t \right)}$$

where

4 $$S\left( t \right) = \mathop \smallint \limits_{t}^{\infty } f\left( y \right)dy = P\left( {T > t} \right) = 1 - F\left( t \right)$$

5 $$F\left( t \right) = \mathop \smallint \limits_{0}^{t} f\left( y \right)dy = P\left( {T < t} \right)$$

The F(t) is the cumulative probability that a woman has her second birth before time *t*; f(t) is the probability density function of the survival time *T*, defined as the probability that a woman has her second childbirth per unit time in a short interval expressed as:

6 $$f\left( t \right) = \mathop {{\text{lim}}}\limits_{\Delta t \to 0} \left\{ {\frac{{P\left( {t \le T < t + \Delta t} \right)}}{\Delta t}} \right\}$$

If *n*_*i*_ is the number of women who were exposed to the risk of having second birth, censored women inclusive, before *i*th survival time *(t*_*i*_*)* and *l*_*i*_ is the number of women who had second birth at *t*_*i*_, then Eq. (7) below estimates the survival functions.

7 $$s\left( t \right) = \mathop \prod \limits_{i = 1}^{m} \left\{ {\frac{{n_{i} - l_{i} }}{{n_{i} }}} \right\} \mathrel\backepsilon t_{m} < t < t_{m + 1} ;\quad s\left( t \right) = 1\,if\,t < t_{1}$$

where* m* is the number of different failure times (i.e., experiencing second birth).

The unadjusted CPH model was used to explain the association between each of the main independent variables including the other covariates and SBI. Using the Wald test to assess the significance of the interplay between the key variables (contraceptive use and fertility desire), the statistically significance of the interaction term was not uniform across the studied countries (this was not presented). Thus, having confirmed non-violation of the proportional hazard assumption, two adjusted CPH models were fitted. Model 1 constitutes only the key independent variables and model 2 includes all the significant variables (*p* < 0.15) based on the log-rank test in addition to the main independent variables. The Wald test using the deviance statistic, − 2log likelihood (− 2LL), was used to select the best model with the least value being adjudged as more adequate.

The hazard ratios (HR) including their 95% confidence intervals are reported. The exponentials of the coefficients (b_j_ which indicates the changes in the expected time to second birth due to a unit change in the jth predictor) suggest the tendency of hazard to second birth; thus, HR > 1 indicates higher hazard and HR < 1 lower hazard. The data was weighted to adjust for differences in population sizes of each region of the selected countries. All analyses were carried out at a 5% level of significance, using STATA 14 SE.

### Ethical considerations

Ethical approval for the parent study was obtained from the National Ethics Committee in the respective countries and the ICF Institutional Review Board. The details of the ethical approval have been reported earlier [32–35]. The present study analysis utilised a secondary dataset, freely available for use in the public domain, which requires no ethics approvals. Meanwhile, the Demographic and Health Surveys Program authorised the utilisation of the dataset for the present analysis.

## Results

### Women’s background characteristics

The distribution of participants and summary statistics of median time to second birth by the selected characteristics are presented in Tables 1 and 2. The women’s mean (± standard deviation) age was 31.1 (± 8.6) in DR Congo; 31.9 (± 8.0), Ethiopia; 32.6 (± 8.5), Nigeria; and 33.6 (± 8.5), South Africa. Teenage mothers at first birth constituted the highest percentage of the participants, ranged from 62.6% (Ethiopia) to 47.3% (South Africa). Most women in DR Congo (67.8%), Ethiopia (73.4%), and Nigeria (62.6%) were rural residents, while less than half lived in rural South Africa. Women in Ethiopia (60.3%) had the highest proportion of no formal education, with South Africa having the lowest (2.6%). While half of South Africa (48.9%) women were never married, nearly all the women in other selected countries were ever married (Tables 1, 2).

**Table 1:** Distribution of women’s characteristics, and their association with the median time to second birth in DR Congo and Ethiopia

Characteristic	DR Congo			Ethiopia		
	n (%)	% SB	MtSb (CI)	n (%)	% SB	MtSb (CI)
*Fertility desired*			0.001***			< .001***
No more	4014(28.9)	95.7	34(33–34)	3897(38.5)	92.7	39(38–40)
Wanted	9191(66.2)	75.0	34(33–34)	5734(56.7)	71.7	45(43–46)
Undecided	679(4.9)	86.7	34(32–35)	483(4.8)	85.5	39(37–42)
*Contraceptive use*			0.289			< .001***
Never	9180(66.1)	81.2	33(33–34)	5138(50.8)	83.0	36(35–37)
Formal	2192(15.8)	83.3	34(34–35)	2103(20.8)	80.0	48(46–50)
Current	2512(18.1)	81.4	35(34–35)	2873(28.4)	76.3	53(51–55)
*Contraception/desire*			< .001***			< .001***
Never/no-more	2574(18.5)	94.9	33(32–34)	1863(18.4)	92.8	36(35–37)
Never/want	6102(43.9)	75.0	33(33–34)	2996(29.6)	76.7	36(35–37)
Never/undecided	504(3.6)	85.5	34(32–36)	279(2.8)	85.3	36(34–39)
Former/no-more	651(4.7)	96.3	34(33–35)	881(8.7)	92.4	43(41–45)
Former/want	1443(10.4)	76.8	34(33–35)	1122(11.1)	70.3	54(51–58)
Former/undecided	98(0.7)	91.8	36(32–40)	100(1.0)	80.0	46(40–66)
Current/no-more	789(5.7)	97.8	35(33–36)	1153(11.4)	92.9	43(41–46)
Current/want	1646(11.9)	73.3	35(34–36)	1616(16.0)	63.5	65(60–70)
Current/undecided	77(0.6)	88.3	30(26–34)	104(1.0)	91.3	44(38–53)
*Ethnicity++*			< .001***			< .001***
Bakongo Nod and Sud	1131(8.2)	77.7	39(37–41)			
Bas-Kasai et Kwilu-Kwngo	2195(15.8)	81.0	35(34–36)			
Cuvette central	1378(9.9)	81.1	34(33–35)			
Ubangi et Itimbiri	1712(12.3)	81.1	33(32–34)			
Uele Lac Albert	1255(9.0)	78.0	33(32–35)			
Basele-K, Man. et Kivu	2265(16.3)	83.7	32(32–33)			
Kasai, Katanga, Tanganika	3690(26.6)	83.2	32(32–33)			
Lunda/others	247(1.8)	82.6	37(34–39)			
Amhara				2189(21.7)	74.4	59(57–61)
Oromo				2352(23.3)	81.8	39(37–40)
Tigrie				1189(11.8)	77.6	50(48–53)
Affar				708(7.0)	82.5	32(30–34)
Somalie				1014(10.0)	87.7	28(28–30)
Guragie/others				2651(26.2)	82.4	41(40–42)
*Residence*			< .001***			< .001***
Urban	4471(32.2)	78.4	36(35–37)	2694(26.6)	68.9	64(61–68)
Rural	9413(67.8)	83.0	33(32–33)	7420(73.4)	84.7	38(37–38)
*Religion+*			0.102			< .001***
Christianity	13,236(95.6)	81.3	34(33–34)	5654(55.9)	77.5	49(48–50)
Islam	225(1.6)	84.9	34(30–36)	4328(42.8)	84.2	35(34–35)
Traditionalist/other	386(2.8)	88.3	33(31–34)	132(1.3)	86.4	35(33–40)
*Age at FB*			< .001***			< .001***
< 20	8417(60.6)	82.5	34(33–34)	6336(62.6)	85.9	40(39–41)
20–24	4247(30.6)	81.8	33(33–34)	2904(28.7)	74.6	44(43–46)
≥ 25	1220(8.8)	73.9	37(35–38)	874(8.6)	60.6	53(47–61)
*Wealth index*			< .001***			< .001***
Low	5316(38.3)	81.6	33(33–34)	4088(40.4)	85.2	35(35–36)
Middle	4658(33.5)	84.1	33(32–33)	2287(22.6)	85.1	40(39–41)
High	3910(28.2)	78.4	36(35–37)	3739(37.0)	72.5	57(54–59)
*Education*			< .001***			< .001***
No education	2952(21.3)	87.6	33(33–34)	6097(60.3)	90.4	36(36–37)
Primary	5863(42.2)	85.4	33(32–33)	2688(26.6)	70.0	53(51–56)
Secondary	4835(34.8)	74.4	35(34–36)	856(8.5)	57.2	87(72–105)
Higher	234(1.7)	56.8	47(38–52)	473(4.7)	53.9	111(82–144)
*Employment+*			0.107			< .001***
Not working	3231(23.3)	74.5	34(33–35)	6491(64.2)	81.8	39(38–40)
Working	10,616(76.5)	83.7	34(33–34)	3623(35.8)	78.1	47(46–48)
*Marital at FB*			< .001***			< .001***
Never married	793(5.7)	27.9	70(60–86)	120(1.2)	26.7	na
Married before FB	11,673(84.1)	84.1	33(33–33)	9254(91.5)	80.3	41(41–42)
Married after FB	1418(10.2)	90.1	35(34–36)	740(7.3)	91.2	40(38–43)
*FB sex*			0.628			0.029*
Male	6959(50.1)	81.4	34(33–34)	5259(52.0)	79.5	42(41–43)
Female	6925(49.9)	81.7	34(33–34)	4855(48.0)	81.5	41(40–43)
*FB survival*			0.491			0.017*
Dead	951(6.8)	77.8	30(29–32)	503(5.0)	80.5	34(32–38)
Alive	12,933(93.2)	81.8	34(33–34)	9611(95.0)	80.5	42(41–43)
Total	13,884	81.5	34(33–34)	10,114	80.5	42(41–42)

***p < .001; **p < .01, *p < .05 (based on log-rank test); + missing (for only Ethiopia); +  + missing not reported; CI—95% Confidence interval; n—number of women; FB—first birth; SB—second birth; MtSb—Median survival time to second childbirth in months; na—not available owing to non-computation of the standard error of the estimated MtSb

**Table 2:** Distribution of women’s characteristics, and their association with the median time to second birth in Nigeria and South Africa

Characteristic	Nigeria			South Africa		
	n (%)	% SB	MtSb (CI)	n (%)	% SB	MtSb (CI)
*Fertility desired*			0.046*			< .001***
No more	9047(30.9)	97.8	36(35–37)	3877(64.2)	80.1	64(62–66)
Wanted	18,283(62.4)	76.6	34(34–35)	1828(30.3)	39.8	105(96–115)
Undecided	1966(6.7)	90.9	35(34–36)	334(5.5)	54.8	83(74–93)
*Contraceptive use*			< .001***			< .001***
Never	19,898(67.9)	82.5	35(34–35)	1350(22.4)	64.5	78(73–84)
Formal	4643(15.8)	86.6	37(36–37)	1295(21.4)	66.8	72(68–76)
Current	4755(16.2)	88.5	35(34–36)	3394(56.2)	67.1	69(67–71)
*Contraception/desire*			< .001***			< .001***
Never/no-more	5377(18.4)	96.8	35(35–36)	872(14.4)	75.5	68(63–72)
Never/want	13,267(45.3)	76.1	34(34–35)	357(5.9)	42.6	121(99–139)
Never/undecided	1254(4.3)	89.4	34(33–35)	121(2.0)	50.4	97(84–124)
Former/no-more	1649(5.6)	98.8	38(37–39)	783(13.0)	82.8	63(59–66)
Former/want	2626(9.0)	78.2	36(35–37)	447(7.4)	40.0	124(98–143)
Former/undecided	368(1.3)	91.3	37(35–39)	65(1.1)	58.5	95(70–116)
Current/no-more	2021(6.9)	99.8	36(35–38)	2222(36.8)	81.0	63(60–65)
Current/want	2390(8.2)	77.9	34(33–35)	1024(17.0)	38.7	93(82–103)
Current/undecided	344(1.2)	95.9	36(33–38)	148(2.5)	56.8	71(62–82)
*Ethnicity*			< .001***			0.003**
Hausa	11,048(37.7)	87.6	33(33–33)			
Igbo	4220(14.4)	82.1	34(33–34)			
Yoruba	3619(12.4)	81.3	41(40–42)			
Don’t know/others	10,409(35.5)	82.3	36(36–37)			
Black/African				5259(87.1)	65.8	72(70–74)
White				131(2.2)	77.9	50(37–60)
Coloured				594(9.8)	69.0	74(67–77)
Indian/Asian/other				55(0.9)	74.5	69(49–87)
*Residence*			< .001***			< .001***
Urban	10,970(37.4)	82.9	36(36–37)	3373(55.9)	64.3	76(74–78)
Rural	18,326(62.6)	84.9	34(34–35)	2666(44.1)	69.3	66(64–69)
*Religion*			< .001***			
Christianity	13,444(45.9)	80.9	37(37–37)			
Islam	15,582(53.2)	87.0	34(33–34)			
Traditionalist/other	270(0.9)	84.4	33(31–35)			
*Age at FB*			< .001***			< .001***
< 20	16,601(56.7)	87.8	35(34–35)	2857(47.3)	71.5	71(69–74)
20–24	8509(29.0)	81.6	35(35–36)	2293(38.0)	65.9	70(68–73)
≥ 25	4186(14.3)	74.8	36(35–37)	889(14.7)	52.0	77(70–84)
*Wealth index *			< .001***			< .001***
Low	10,208(34.8)	86.4	34(33–34)	2168(35.9)	69.2	64(62–67)
Middle	10,313(35.2)	84.5	35(35–35)	2139(35.4)	66.6	72(69–75)
High	8775(30.0)	81.0	37(37–38)	1732(28.7)	63.0	80(77–85)
*Education*			< .001***			< .001***
No education	12,207(41.7)	89.0	33(33–34)	158(2.6)	87.3	55(50–62)
Primary	5210(17.8)	89.9	36(35–37)	697(11.5)	83.8	58(55–63)
Secondary	9319(31.8)	76.7	37(36–37)	4547(75.3)	64.3	74(71–76)
Higher	2560(8.7)	76.3	37(36–38)	637(10.5)	57.8	78(71–84)
*Employment*			< .001***			< .001***
Not working	8172(27.9)	78.6	33(33–34)	3702(61.3)	63.4	67(66–70)
Working	21,124(72.1)	86.3	36(35–36)	2337(38.7)	71.4	77(75–80)
*Marital at FB*			< .001***			< .001***
Never married	903(3.1)	32.2	91(83–104)	2956(48.9)	52.3	82(79–85)
Married before FB	25,025(85.4)	84.9	34(34–35)	1297(21.5)	77.6	60(58–62)
Married after FB	3368(11.5)	92.6	37(37–38)	1786(29.6)	81.8	69(66–73)
*FB sex*			0.676			0.965
Male	15,019(51.3)	84.0	35(35–35)	3138(52.0)	66.4	71(69–74)
Female	14,277(48.7)	84.2	35(35–35)	2901(48.0)	66.5	72(69–75)
*FB survival*			< .001***			0.488
Dead	1958(6.7)	85.6	31(31–32)	219(3.6)	69.4	64(56–83)
Alive	27,338(93.3)	84.0	35(35–35)	5820(96.4)	66.4	71(70–74)
Total	29,296	84.1/0!	35(35–35)	6039	66.5	71(70–73)

***p < .001; **p < .01, *p < .05 (based on log-rank test); CI—95% Confidence interval; n—number of women; FB—first birth; SB—second birth; MtSb—Median survival time to second childbirth in months

The percentage distribution of fertility desire and contraceptive use were similar in Nigeria and DR Congo. About two-thirds of the women either desired additional child(ren) or never used contraceptive in each of these countries. Only 30.3% of the women desired additional child(ren) and 22.4% never used contraceptive in South Africa; half never used contraceptive (50.8%) and nearly three-fifths desired additional child (56.7%) in Ethiopia. Most women desired additional child(ren) but never use contraceptive in Nigeria (45.3%), DR Congo (43.9%), Ethiopia (29.6%); but few, in South Africa (5.9%) (Tables 1, 2).

More than 80% of the women in DR Congo (81.5%), Ethiopia (80.5%) and Nigeria (84.1%), compared to only three-quarters in South Africa (66.5%), had second birth as of the survey date (Tables 1, 2).

### The pattern of SBI by fertility desire and contraceptive use

**Fig. 1:**
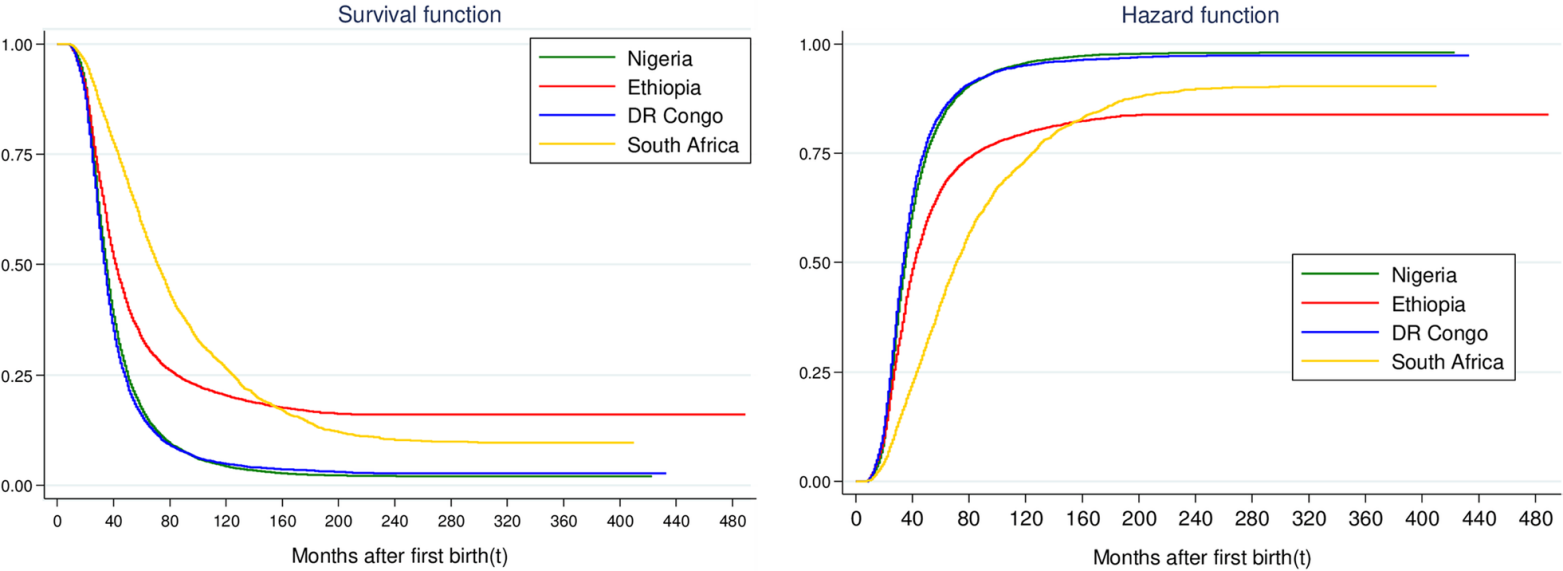
Overall survival and hazard functions of second birth interval. The probability plot showing the cumulative survival curve and the risk of having a second birth after the first birth by selected countries

**Fig. 2:**
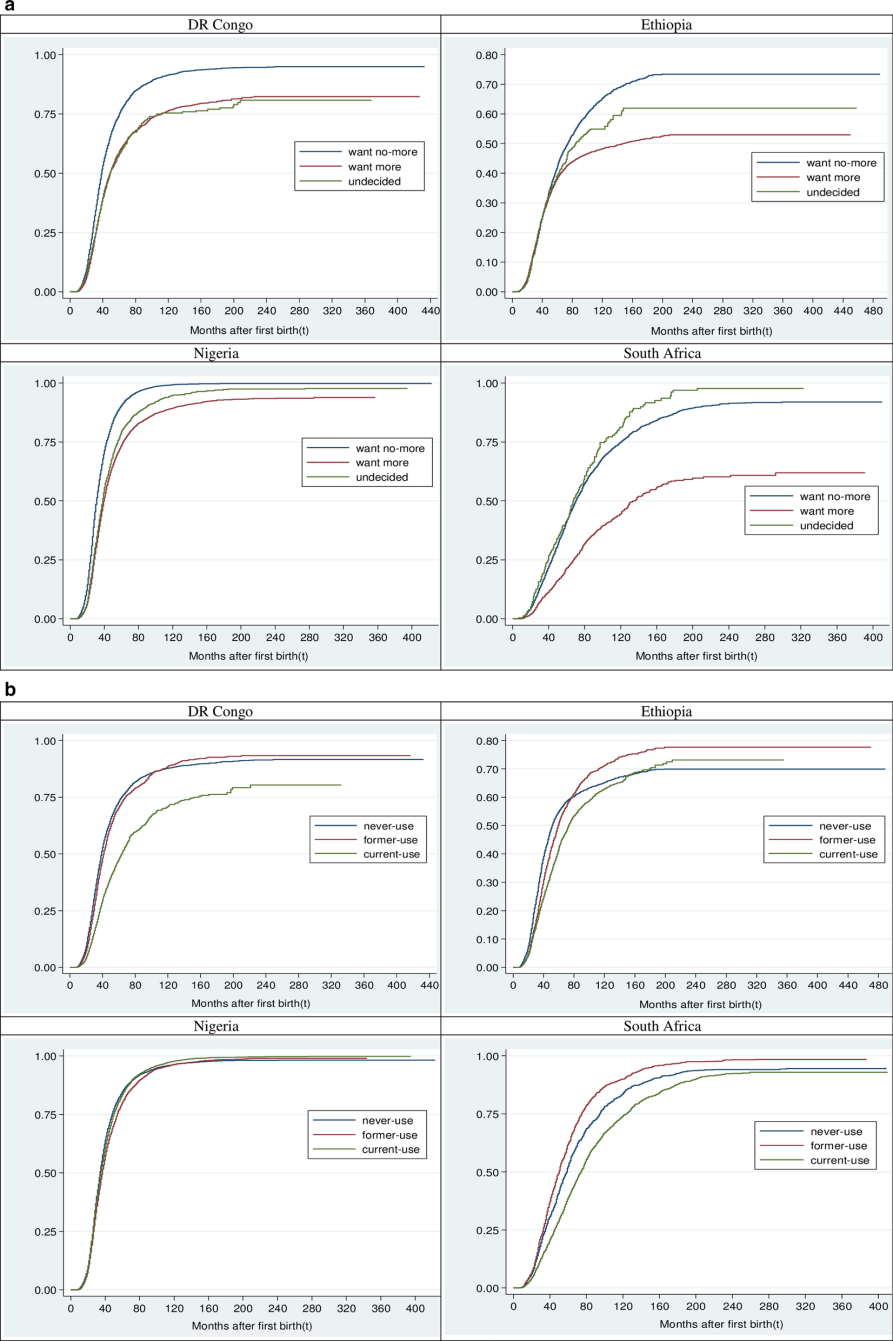

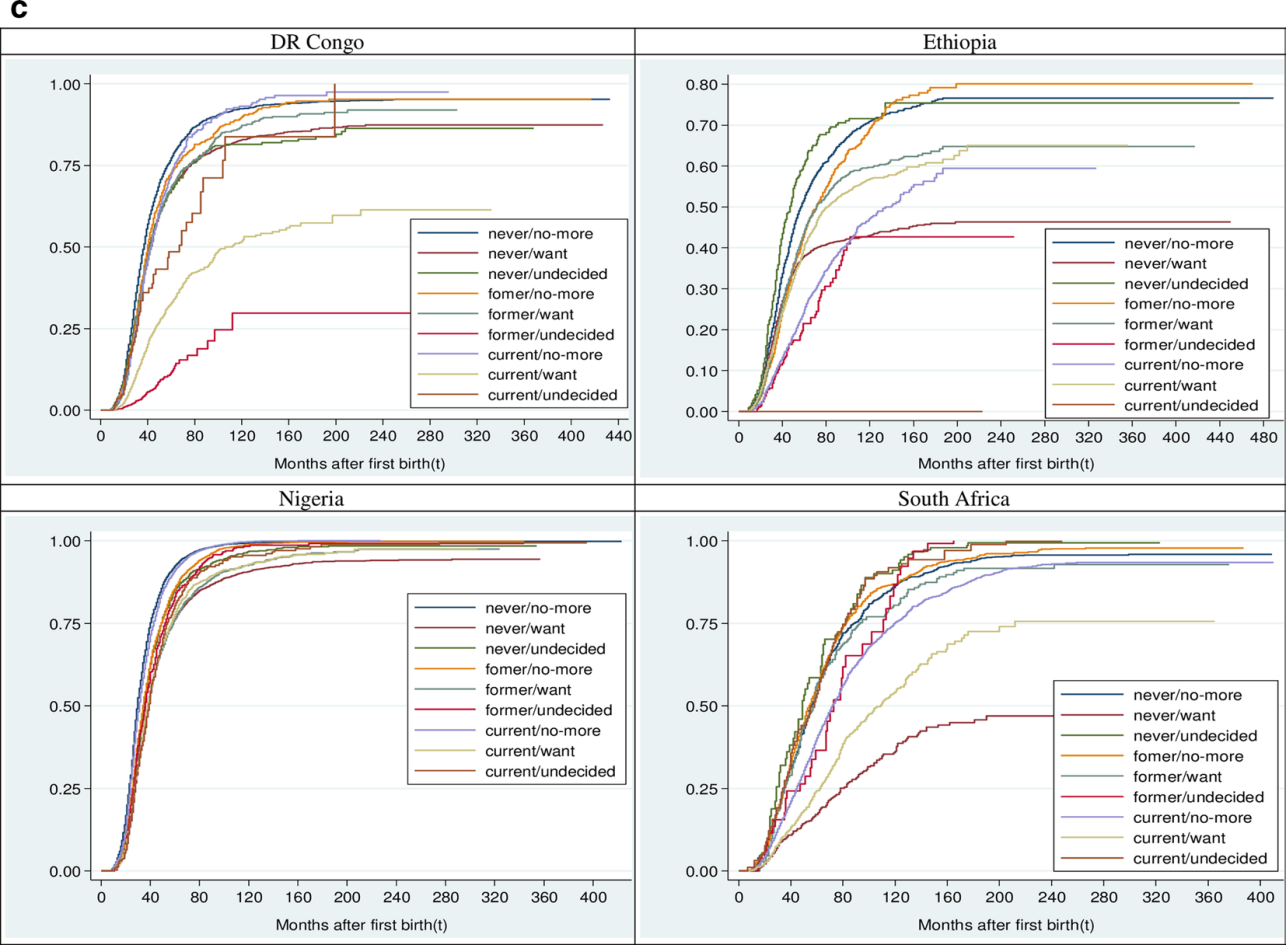
Hazard function of time to second birth by **a** fertility desire in the selected countries. A graphical presentation of the comparison of probability of having a second childbirth by fertility desire. **b** Contraceptive use in the selected countries. A graphical display of the comparison of probability of having a second childbirth by contraception. **c** Fertility desire and contraceptive use interplay in the selected countries. A graphical plot of the comparison of probability of having a second childbirth by interaction between fertility desire and contraceptive use

The SBI duration did not vary considerably by fertility desire nor contraception in Nigeria and DR Congo. The two countries had relatively similar duration of SBI among women who desired another child (Nigeria, 34 (34–35); DR Congo, 34 (33–34) months) or never used contraceptive (Nigeria, 35 (34–35); DR Congo, 33 (33–34) months). In the other two countries, the median time to second birth was longer among women who desired additional child (Ethiopia, 45(43–46); South Africa, 105(96–115) months) compared to those who wanted no more. However, relative to women who currently used contraceptive, shorter and longer SBI durations were respectively observed among Ethiopian (36 vs 53 months) and South African (78 vs 69 months) women who never used contraceptive. By and large, the median time to second birth was 34 (33–34) months in DR Congo; 35(35–35) months, Nigeria; 42 (41–42) months, Ethiopia; and 71 (70–73) months, South Africa (Tables 1, 2). This result is also corroborated by Fig. 1, which reveals the cumulative survival rate along with the probability of the risk of having a second birth by selected countries. In addition, Fig. 2a–c show the probabilities of having second birth by fertility desire, contraceptive use and their interplay in these countries. Almost all the selected variables had significant (*p* < 0.15) differences in their respective survival curves category except for sex (DR Congo, Nigeria and South Africa) and the survival status of the first child (DR Congo and South Africa).

### Influence of fertility desire and contraception on time to second childbirth

The outcomes of unadjusted and adjusted CPH regression models to investigate the contribution of fertility desire and contraception to the duration of SBI are presented in Tables 3, 4, 5. The association of fertility desire, including the use of contraceptive, with the duration of SBI varied across the considered countries. The association remained similar even after respectively controlling for contraceptive use and other included variables. Women in DR Congo (aHR = 0.93; CI: 0.89–0.97) and Ethiopia (aHR = 0.64; CI: 0.61–0.67) who desired additional child(ren) were 7% and 36% more likely to delay a second birth, whereas those in South Africa (aHR = 0.51; CI: 0.47–0.55) were about 50% more likely compared with those who wanted no more children (Tables 3, 4). In Table 5, irrespective of the affiliated country, hazard of second birth is lower among women who desired additional child(ren).

**Table 3:** Factors associated with time to second birth interval among women in DR Congo and Ethiopia

Characteristic	DR Congo			Ethiopia		
	Crude model	Model 1	Model 2	Crude model	Model 1	Model 2
	HR (95% CI)	aHR (95% CI)	aHR (95% CI)	HR (95% CI)	aHR (95% CI)	aHR (95% CI)
*Fertility desired*						
No more (R)	1	1	1	1	1	1
Wanted	0.93(0.89,0.96)^c^	0.93(0.89,0.96)^c^	0.93(0.89,0.97)^c^	0.64(0.62,0.67)^c^	0.64(0.61,0.67)^c^	0.64(0.61,0.67)^c^
Undecided	0.92(0.84,1.00)	0.92(0.84,1.00)^a^	0.92(0.84,1.00)	0.87(0.78,0.96)^b^	0.84(0.76,0.93)^c^	0.80(0.72,0.89)^c^
*Contraceptive use*						
Never	1.03(0.98,1.08)	1.03(0.98,1.09)	0.88(0.84,0.93)^c^	1.46(1.38,1.53)^c^	1.45(1.37,1.52)^c^	1.02(0.96,1.08)
Formal	1.00(0.94,1.06)	1.00(0.94,1.07)	0.92(0.86,0.98)^b^	1.08(1.02,1.15)^a^	1.06(1.00,1.13)	1.02(0.95,1.08)
Current (R)	1	1	1	1	1	1
*Ethnicity*						
Bakongo Nod&Sud (R)	1		1			
Bas-Kasai et Kwilu-Kwngo	1.20(1.10,1.30)^c^		1.10(1.01,1.20)^a^			
Cuvette central	1.26(1.15,1.38)^c^		1.12(1.02,1.23)^a^			
Ubangi et Itimbiri	1.35(1.24,1.46)^c^		1.21(1.11,1.33)^c^			
Uele Lac Albert	1.20(1.10,1.32)^c^		1.09(0.99,1.20)			
Basele-K, Man. et Kivu	1.45(1.33,1.57)^c^		1.33(1.22,1.44)^c^			
Kasai, Katanga,Tanganika	1.49(1.38,1.60)^c^		1.33(1.23,1.44)^c^			
Lunda/others	1.22(1.05,1.42)^a^		1.14(0.98,1.33)			
Amhara (R)				1		1
Oromo				1.51(1.41,1.61)^c^		1.38(1.29,1.49)^c^
Tigrie				1.15(1.06,1.24)^b^		1.17(1.08,1.27)^c^
Affar				1.88(1.71,2.06)^c^		1.39(1.24,1.56)^c^
Somalie				2.31(2.12,2.50)^c^		1.91(1.73,2.11)^c^
Guragie/others				1.46(1.37,1.56)^c^		1.44(1.35,1.54)^c^
*Residence*						
Urban (R)	1		1	1		1
Rural	1.28(1.23,1.33)^c^		1.15(1.09,1.22)^c^	1.82(1.73,1.92)^c^		1.21(1.12,1.30)^c^
*Religion*						
Christianity (R)	1		1	1		1
Islam	1.08(0.93,1.24)		0.98(0.84,1.13)	1.50(1.44,1.57)^c^		1.15(1.08,1.21)^c^
Traditionalist/other	1.11(1.00,1.24)		1.04(0.94,1.16)	1.54(1.28,1.85)^c^		1.23(1.01,1.48)^a^
*Age at FB*						
< 20 (R)	1		1	1		1
20–24	1.04(0.99,1.08)		1.06(1.01,1.10)^b^	0.77(0.74,0.81)^c^		0.88(0.84,0.93)^c^
≥ 25	0.78(0.73,0.84)^c^		0.82(0.77,0.88)^c^	0.56(0.51,0.61)^c^		0.73(0.66,0.79)^c^
*Wealth index *						
Low (R)	1		1	1		1
Middle	1.04(0.99,1.08)		1.03(0.99,1.08)	0.87(0.82,0.92)^c^		0.99(0.93,1.05)
High	0.80(0.76,0.83)^c^		0.97(0.91,1.04)	0.57(0.54,0.60)^c^		0.95(0.88,1.02)
*Education*						
No education (R)	1		1	1		1
Primary	1.07(1.02,1.12)^b^		1.08(1.03,1.14)^b^	0.53(0.50,0.56)^c^		0.63(0.60,0.67)^c^
Secondary	0.85(0.80,0.89)^c^		0.96(0.90,1.02)	0.37(0.33,0.40)^c^		0.53(0.48,0.58)^c^
Higher	0.49(0.41,0.58)^c^		0.71(0.59,0.85)^c^	0.33(0.29,0.37)^c^		0.53(0.46,0.61)^c^
*Employment*						
Not working (R)	1		1	1		1
Working	1.04(0.99,1.09)		0.98(0.93,1.02)	0.81(0.77,0.85)^c^		0.95(0.90,0.99)^a^
*Marital at FB*						
Never married (R)	1		1	1		1
Married before FB	3.30(2.88,3.77)^c^		2.88(2.52,3.30)^c^	5.43(3.84,7.68)^c^		3.94(2.78,5.58)^c^
Married after FB	2.72(2.36,3.14)^c^		2.49(2.16,2.88)^c^	6.47(4.54,9.23)^c^		4.42(3.09,6.31)^c^
*FB sex*						
Male (R)	1			1		1
Female	1.01(0.97,1.05)			1.05(1.00,1.10)^a^		1.06(1.02,1.11)^b^
*FB survival*						
Dead	0.97(0.90,1.05)			1.13(1.02,1.25)^a^		1.02(0.92,1.12)
Alive (R)	1			1		1
*Deviance (−2ll)*		*193,721.0*^b^	*191,553.4*^c^		*139,264.8*^c^	*137,602.6*^c^

^c^*p* < .001; ^b^*p* < .01, ^a^*p* < .05; (a)HR—(adjusted) hazard ratio; CI—confidence interval; R—reference category; ll—loglikelihood; FB—first birth

**Table 4:** Factors associated with time to second birth interval among women in Nigeria and South Africa

Characteristic	Nigeria			South Africa		
	Crude model	Model 1	Model 2	Crude model	Model 1	Model 2
	HR (95% CI)	aHR (95% CI)	aHR (95% CI)	HR (95% CI)	aHR (95% CI)	aHR (95% CI)
*Fertility desired*						
No more (R)	1	1	1	1	1	1
Wanted	1.02(0.99,1.05)	1.02(0.99,1.05)	1.00(0.97,1.03)	0.50(0.46,0.54)^c^	0.51(0.47,0.55)^c^	0.51(0.47,0.55)^c^
Undecided	1.07(1.01,1.12)^a^	1.07(1.01,1.12)^a^	1.03(0.98,1.08)	0.73(0.62,0.84)^c^	0.76(0.65,0.88)^c^	0.74(0.64,0.86)^c^
*Contraceptive use*						
Never	0.97(0.93,1.00)	0.96(0.93,1.00)^a^	0.80(0.77,0.83)^c^	0.75(0.69,0.81)^c^	0.77(0.71,0.83)^c^	0.76(0.70,0.82)^c^
Formal	0.88(0.84,0.92)^c^	0.88(0.84,0.92)^c^	0.84(0.81,0.88)^c^	0.85(0.79,0.92)^c^	0.91(0.84,0.98)^a^	0.90(0.83,0.97)^b^
Current (R)	1	1	1	1	1	1
*Ethnicity*						
Hausa (R)	1		1			
Igbo	0.78(0.75,0.81)^c^		1.06(1.00,1.13)^a^			
Yoruba	0.62(0.60,0.65)^c^		0.77(0.73,0.81)^c^			
Don’t know/others	0.73(0.71,0.75)^c^		0.89(0.85,0.92)^c^			
Black/African (R)				1		1
White				1.45(1.19,1.76)^c^		1.53(1.24,1.88)^c^
Coloured				0.99(0.89,1.10)		1.05(0.94,1.17)
Indian/Asian/other				1.00(0.74,1.36)		1.09(0.80,1.49)
*Residence*						
Urban (R)	1		1	1		1
Rural	1.16(1.13,1.19)^c^		1.05(1.02,1.09)^b^	1.27(1.19,1.35)^c^		1.16(1.07,1.24)^c^
*Religion*						
Christianity (R)	1		1			
Islam	1.37(1.33,1.40)^c^		1.22(1.17,1.27)^c^			
Traditionalist/other	1.17(1.03,1.34)^a^		1.31(1.14,1.49)^c^			
*Age at FB*						
< 20 (R)	1		1	1		1
20–24	0.92(0.89,0.95)^c^		1.02(0.99,1.05)	0.99(0.92,1.06)		1.02(0.96,1.10)
≥ 25	0.83(0.80,0.87)^c^		0.98(0.94,1.02)	0.80(0.72,0.88)^c^		0.88(0.79,0.98)^a^
*Wealth index*						
Low (R)	1		1	1		1
Middle	0.86(0.83,0.88)^c^		0.95(0.92,0.98)^b^	0.87(0.81,0.94)^c^		0.92(0.86,1.00)^a^
High	0.73(0.71,0.75)^c^		0.87(0.83,0.90)^c^	0.71(0.66,0.77)^c^		0.75(0.68,0.83)^c^
*Education*						
No education (R)	1		1	1		1
Primary	0.81(0.79,0.84)^c^		0.97(0.93,1.01)	0.97(0.81,1.17)		0.96(0.79,1.15)
Secondary	0.75(0.73,0.77)^c^		0.99(0.95,1.03)	0.74(0.63,0.88)^b^		0.86(0.72,1.02)
Higher	0.69(0.66,0.72)^c^		0.93(0.87,0.99)^a^	0.67(0.55,0.81)^c^		0.88(0.71,1.08)
*Employment*						
Not working (R)	1		1	1		1
Working	0.85(0.82,0.87) ^c^		0.95(0.92,0.98)^b^	0.85(0.79,0.90)^c^		0.85(0.79,0.90)^c^
*Marital at FB*						
Never married (R)	1		1	1		1
Married before FB	3.67(3.27,4.12)^c^		3.26(2.90,3.66)^c^	1.63(1.51,1.77)^c^		1.57(1.45,1.70)^c^
Married after FB	3.02(2.68,3.41)^c^		2.87(2.55,3.24)^c^	1.30(1.21,1.39)^c^		1.32(1.23,1.42)^c^
*FB sex*						
Male (R)	1			1		
Female	1.00(0.97,1.02)			1.00(0.94,1.07)		
*FB survival*						
Dead	1.13(1.07,1.19)^c^		1.11(1.05,1.16)^c^	0.94(0.80,1.11)		
Alive (R)	1		1	1		
*Deviance (-2ll)*		*458,619.0*^c^	*456,902.2*^c^		*62,423.8*^c^	*62,116.4*^c^

^c^*p* < .001; ^b^*p* < .01, ^a^*p* < .05; (a)HR—(adjusted) hazard ratio; CI–confidence interval; R—reference category; ll—loglikelihood; FB—first birth

**Table 5:** Crude and adjusted hazard ratios of time to second birth interval among women in selected four SSA countries

Characteristics	Crude model	Model 1	Model 2
	HR (95% CI)	aHR (95% CI)	aHR (95% CI)
*Fertility desired*			
No more (R)	1	1	1
Wanted	0.96 (0.94,0.98)***	0.93 (0.92,0.95)***	0.86 (0.85,0.88)***
Undecided	1.04 (1.00,1.08)	1.00 (0.96,1.04)	0.92 (0.89,0.96)***
*Contraceptive use*			
Never	1.34 (1.31,1.37)***	1.35 (1.32,1.38)***	0.93 (0.90,0.95)***
Formal	1.07 (1.04,1.10)***	1.07 (1.04,1.10)***	0.93 (0.90,0.95)***
Current (R)	1	1	1
*Country*			
Nigeria	2.50 (2.42,2.59)***		1.85 (1.78,1.93)***
Ethiopia	1.47 (1.42,1.53)***		0.99 (0.95,1.04)
DR Congo	2.64 (2.54,2.73)***		2.01 (1.93,2.10)***
South Africa (R)	1		1
*Residence*			
Urban (R)	1		1
Rural	1.35 (1.32,1.37)***		1.10 (1.07,1.13)***
*Age at birth*			
< 20 (R)	1		1
20–24	0.89 (0.87,0.90)***		0.98 (0.96,1.00)*
≥ 25	0.77 (0.75,0.79)***		0.87 (0.84,0.90)***
*Wealth index*			
Low (R)	1		1
Middle	0.92 (0.90,0.94)***		0.96 (0.94,0.99)**
High	0.70 (0.69,0.72)***		0.86 (0.83,0.88)***
*Education*			
No education (R)	1		1
Primary	0.81 (0.79,0.82)***		0.84 (0.82,0.86)***
Secondary	0.65 (0.64,0.67)***		0.81 (0.79,0.83)***
Higher	0.56 (0.54,0.59)***		0.75 (0.72,0.79)***
*Employment status*			
Not working (R)	1		1
Working	1.12 (1.10,1.14)***		0.91 (0.90,0.93)***
*Marital at FB*			
Never married (R)	1		1
Married before FB	2.87 (2.74,3.00)***		2.13 (2.03,2.24)***
Married after FB	2.25 (2.14,2.36)***		1.84 (1.75,1.93)***
*FB sex*			
Male	1		1
Female (R)	1.02 (1.00,1.04)		1.01 (0.99,1.03)
*FB survival*			
Dead	1.15 (1.10,1.19)***		1.06 (1.02,1.09)**
Alive (R)	1		1
*Deviance (-2ll)*		971,933.6***	964,037.4***

^***^p < .001; **p < .01, *p < .05; (a)HR—(adjusted) hazard ratio; CI—confidence interval; R—reference category; ll—loglikelihood; FB—first birth

On the other hand, the tendency of having a second birth was significantly higher among Ethiopian women who never used contraceptive (aHR = 1.45; CI: 1.37–1.52) even after controlling for fertility desire; however, the hazard became non-significant when other studied variables were adjusted for (Table 3). Relative to women who currently used contraceptive, the risk of having a second birth was significantly lower among those who never used (DR Congo—aHR = 0.88, CI: 0.84–0.93; Nigeria—aHR = 0.80, CI: 0.77–0.83; South Africa—aHR = 0.76, CI: 0.70–0.82) and those who were former users (DR Congo—aHR = 0.92, CI: 0.86–0.98; Nigeria—aHR = 0.81, CI: 0.81–0.88; South Africa—aHR = 0.90, CI: 0.83–0.97) (Tables 3, 4). Overall, never or former users of contraceptives were less likely to delay second birth even after controlling for fertility desire; however, they were more likely to delay it when other covariates were controlled for (Table 5).

Ethnicity, place of residence and marital status at first birth were other significant predictors that influenced the timing of second birth which were comparable among the four countries. For example, the hazard of having a shortened SBI was significantly higher among women residing in rural. However, increasing age at first birth increased the likelihood of lengthening SBI though statistically non-significant in Nigeria. The likelihood of lengthening SBI increased as the household wealth quintile rose in Nigeria and South Africa, and as educational attainment increased in DR Congo and Ethiopia. Additionally, women whose first child were not alive were over 10% times more likely to shorten SBI compared to those whose first child survived in Nigeria. However, the hazard of having second birth was higher among women whose first childbirth were female (aHR = 1.06; CI: 1.02–1.11) compared to male in Ethiopia. Of note, DR Congo and Nigerian women were about two times more likely to shorten SBI compared to their South African counterparts (Table 5).

## Discussion

This study was aimed to explore the impact of contraceptive use and fertility desire on time to second childbirth duration in SSA. The observed median time to second birth in this study suggests that inter-birth interval remains sub-optimally short in DR Congo and Nigeria, long in South Africa and optimal in Ethiopia. Even though the finding indicates relatively optimal SBI, a few studies conducted in Ethiopia have reported a high prevalence of sub-optimally short birth interval [37, 38]. This suggests that birth interval still impacts negatively on maternal and child health in many parts of SSA. The findings further reinforce the need to strengthen context-specific policies and promote optimal birth interval by maternal- and child-care providers and policymakers across SSA countries.

Studies have rarely explored the impact of fertility desire and contraceptive use on SBI. The observed shortest duration of SBI in DR Congo and longest in South Africa relative to their respective fertility rate among the studied countries attest to the submission that the higher the TFR the more shortened the inter-birth interval and vice versa. Relative to DR Congo, nearly equivalent SBI duration was observed among women in Nigeria. A similar finding was reported earlier [16]. The nearly equivalent SBI duration observed among women in DR Congo and Nigeria did not vary considerably by fertility desire nor contraception. However, the finding of relatively lengthened SBI among South Africa including Ethiopian women could be partly attributed to the preference for additional child(ren); this aligns with the previous study [39]. A high percentage of women who had second birth (observed among those who wanted no more child) could suggest that the women have already attained their desired fertility. Hence, the delayed second birth and subsequently, the long duration of SBI in South Africa and relatively in Ethiopia as noted in this study.

Unlike DR Congo, Ethiopia and Nigeria, the prevalence of unmet need for contraceptive was low among women in South Africa (5.9%). The possible explanation could be the high uptake of contraceptive in South Africa in relation to other SSA countries. Other researchers have alluded to these findings [17]. In addition, the tendency to delay a second birth was nearly 50% times less likely among women who never used contraceptive in Ethiopia. The association, though remained after adjusting for fertility desire, statistically became non-significant when other included variables’ likely influence was removed. However, a relatively lower hazard of having a second birth was observed among women who never used contraceptive in DR Congo, Nigeria and South Africa. This contrasts the expectation that contraceptive non-use exposes women to the risk of unwanted second births early, often associated with high parity [39–41]. By and large, one possible explanation of the association between non-use of contraceptive and delayed second birth may be linked to the attainment of the desired fertility. Another possible explanation could be the long duration of breastfeeding practices notably among SSA women [38].

Of note, the effect of fertility desire on SBI remains unchanged even after controlling for contraceptive use alone and other variables including contraceptive use, respectively. The finding suggests that the effect of fertility desire on SBI is independent of other variables’ influence, contraceptive use inclusive. Studies have demonstrated the strong relationship between the desire for more children and birth interval [19, 30]. On the other hand, the effect of contraceptive use on SBI substantially changed, when other variables’ effect was considered including fertility desire. This indicates that the use of contraceptive alone may not have contributed to the relative increase in SBI in SSA [10].

Other determinants of SBI duration include residence, ethnicity and marital status at first birth in all selected countries; others such as age, religion, education, wealth status and employment status, and first birth survival and sex are country-specific predictors. For instance, being a rural resident and a married woman respectively increased the hazard of having second childbirth early; being aged ≥ 25 years increased the tendency of having a lengthened SBI across all the considered countries but Nigeria. This corroborates early literature [16]. Furthermore, being a non-Christian among Ethiopian and Nigerian women was significantly associated with shortened SBI; having an increased wealth quintile among Nigerian and South African women and a higher attained education level among DR Congo and Ethiopian women was strongly related to increased SBI, respectively. While first birth sex was among the important predictors of SBI in Ethiopia, survival of the first child was important in Nigeria.

### Study limitation

A few limitations were observed in this study. One, the study design is cross-sectional in which a causal relationship between the variables and time to second birth cannot be established. Two, there may be a possibility of recall bias due to self-reported data extracted from the original dataset without any means of verification. Nonetheless, the study has been strengthened using a large nationally representative dataset. Besides, the strength of the work includes the provision of information on how fertility desire and contraceptive use impacted the second birth interval in SSA, which is rarely documented.

## Conclusions

Evidence of sub-optimal short and long duration of SBI was observed among women in most sub-regions of SSA. The findings demonstrate country context-specific variation in fertility desires and contraception effect on SBI among women in SSA. While desired for additional child(ren) was strongly associated with SBI in DR Congo, Ethiopia and South Africa; contraceptive non-use was positively related to SBI in Nigeria, Ethiopia and South Africa. Fertility effect on SBI is not only independent of contraceptive use status but also independent of other included important variables. Other predictors—the place of residence, ethnicity, age, marital status at first birth, religion, education, wealth status and employment status, and first birth survival and sex—have also been identified to impact on second birth interval. Relevant policymakers should strengthen and intensify context-specific family planning campaign aimed to address the low uptake of contraceptives and associated challenges of unmet need for contraception. Also, context-specific strategies and policies aimed at containing the associated challenges of densely populated countries through advocacy for low fertility desire should be promoted. These may have a far-reaching impact on women’s fertility desire, contraceptive use, adherence to the optimal inter-birth interval, and consequently improved maternal and child health.

## Abbreviations

SSA: Sub-Saharan African
SBI: Second birth interval
(a)HR: (Adjusted) Hazard ratio
PRB: Population Refence Bureau
TFR: Total fertility rate
DR Congo: Democratic Republic of Congo
DHS: Demographic and Health Survey
DRCDHS: Democratic Republic of Congo
NDHS: Nigeria Demographic and Health Survey
EDHS: Ethiopia Demographic and Health Survey
SADHS: South Africa Demographic and Health Survey
EAs: Enumeration areas
CPH: Cox proportional hazard
LL: Log-likelihood
MtSb: Median survival time to second childbirth in months
FB: First birth
SB: Second birth

## Declarations

### Availability of data and materials

Data sharing does not apply to this article as no new data were created or analysed in this study. The data is publicly available and openly accessible at: https://dhsprogram.com/
